# Classifying Lung Neuroendocrine Neoplasms through MicroRNA Sequence Data Mining

**DOI:** 10.3390/cancers12092653

**Published:** 2020-09-17

**Authors:** Justin J. M. Wong, Paula S. Ginter, Kathrin Tyryshkin, Xiaojing Yang, Jina Nanayakkara, Zier Zhou, Thomas Tuschl, Yao-Tseng Chen, Neil Renwick

**Affiliations:** 1 Laboratory of Translational RNA Biology, Department of Pathology and Molecular Medicine, Queen’s University, Kingston, ON K7L 3N6, Canada; justin.wong@queensu.ca (J.J.M.W.); kt40@queensu.ca (K.T.); xy2@queensu.ca (X.Y.); jina.nanayakkara@queensu.ca (J.N.); 15zz53@queensu.ca (Z.Z.); 2 Department of Pathology and Laboratory Medicine, Weill Cornell Medicine, New York, NY 10065, USA; psg9003@med.cornell.edu (P.S.G.); ytchen@med.cornell.edu (Y.-T.C.); 3 Laboratory of RNA Molecular Biology, The Rockefeller University, New York, NY 10065, USA; ttuschl@mail.rockefeller.edu

**Keywords:** lung neuroendocrine neoplasms, classification, microRNA, markers, small RNA sequencing

## Abstract

**Simple Summary:**

Lung neuroendocrine neoplasms (NENs) are a subset of lung cancer that is difficult to diagnose. MicroRNAs (miRNAs) are small RNA molecules that are valuable markers in many cancers. In this study, we generated miRNA profiles for 55 preserved lung NEN samples (14 typical carcinoid (TC), 15 atypical carcinoid (AC), 11 small cell lung carcinoma (SCLC), and 15 large cell neuroendocrine carcinoma (LCNEC)), and randomly assigned them to either discovery or validation sets. We used machine learning and data mining algorithms to identify important miRNA that can distinguish between the types. Using the miRNAs identified with these algorithms, we were able to distinguish between carcinoids (TC and AC) and neuroendocrine carcinomas (SCLC and LCNEC) in the discovery set with 93% accuracy; in the validation set, we were able to distinguish between these groups with 100% accuracy. Using the same machine learning and data mining techniques, we also identified miRNAs that can distinguish between TC and AC, and SCLC and LCNEC, however more samples are needed to validate these findings.

**Abstract:**

Lung neuroendocrine neoplasms (NENs) can be challenging to classify due to subtle histologic differences between pathological types. MicroRNAs (miRNAs) are small RNA molecules that are valuable markers in many neoplastic diseases. To evaluate miRNAs as classificatory markers for lung NENs, we generated comprehensive miRNA expression profiles from 14 typical carcinoid (TC), 15 atypical carcinoid (AC), 11 small cell lung carcinoma (SCLC), and 15 large cell neuroendocrine carcinoma (LCNEC) samples, through barcoded small RNA sequencing. Following sequence annotation and data preprocessing, we randomly assigned these profiles to discovery and validation sets. Through high expression analyses, we found that miR-21 and -375 are abundant in all lung NENs, and that miR-21/miR-375 expression ratios are significantly lower in carcinoids (TC and AC) than in neuroendocrine carcinomas (NECs; SCLC and LCNEC). Subsequently, we ranked and selected miRNAs for use in miRNA-based classification, to discriminate carcinoids from NECs. Using miR-18a and -155 expression, our classifier discriminated these groups in discovery and validation sets, with 93% and 100% accuracy. We also identified miR-17, -103, and -127, and miR-301a, -106b, and -25, as candidate markers for discriminating TC from AC, and SCLC from LCNEC, respectively. However, these promising findings require external validation due to sample size.

## 1. Introduction

Lung neuroendocrine neoplasms (NENs) are variably aggressive tumors that can be challenging to differentiate based on morphological grounds [1,2,3,4,5]. Lung NENs are subdivided into four pathological types, namely typical carcinoid (TC), atypical carcinoid (AC), large cell neuroendocrine carcinoma (LCNEC), and small cell lung carcinoma (SCLC). Typical carcinoids and atypical carcinoids are well-differentiated low-grade tumors, with the latter carrying a higher malignant potential. In contrast, LCNEC and SCLC are poorly-differentiated high-grade malignant carcinomas [2,3]. Accurate histologic diagnosis is critical as pathological type conveys prognostic information and guides clinical management [4,6,7,8,9]. Although lung NEN classification has been increasingly refined [2], subtle pathological features can result in sample misclassification [1]. Recently, NEN experts called for further research to aid discrimination of lung NEN pathological types [3,10].

MicroRNAs (miRNAs) are small (19–24 nucleotides) RNA molecules that can be used to classify tumor tissues [11]. These regulatory molecules also provide valuable mechanistic insights into tumorigenic processes, through predictable targeting of messenger RNAs [12]. Based on their widespread utility in cancer molecular diagnostics [13], we and others hypothesized that miRNAs could be useful adjunct tissue markers for classifying lung NENs [14,15,16,17,18,19]. Some concerns have been expressed about the variability of miRNA clinical testing [20]; however their stability in fresh and archived tissue [21], in addition to advances in quantitative miRNA detection [22,23], small RNA sequence annotation and genomic organization [24], and machine learning [25] readily support using miRNAs to classify NENs.

Here, we assess miRNA-based classification of lung NENs using a machine learning approach [25]. Through high expression analyses, we identified miRNA tissue markers that are common to all lung NENs. Leveraging prior knowledge that carcinoids (TC and AC) and NECs (SCLC and LCNEC) have major clinical, epidemiologic, histologic, and genetic differences [2], we constructed a classifier that discriminates carcinoids from NECs. We also identified candidate miRNA markers for discriminating TC and AC, as well as SCLC and LCNEC.

## 2. Materials and Methods

### 2.1. Clinical Materials and Study Design

Lung NEN cases (14 TC, 15 AC, 11 SCLC, and 15 LCNEC) were identified in the Department of Pathology and Laboratory Medicine, Weill Cornell Medicine. Hematoxylin-eosin-stained tissue sections from each case were reviewed by experienced pathologists (Paula S. Ginter, Yao-Tseng Chen) using the WHO classification of lung tumors [26]. Slides were reviewed and mitoses were counted using an Olympus microscope, with a 40× objective, and with a field diameter of 0.55 in 6 mm^2^ of viable tumor (25 HPF), and the average mitotic figure per 2 mm^2^ was calculated [27]. Slides were scanned in a routine manner and areas of highest density staining were located. Using an Olympus microscope, with a 40× objective, one author (Paula S. Ginter) manually counted a minimum of 2000 tumor cells to calculate the Ki-67 labeling index [28]. Positive nuclear staining of tumor cells under the microscope was of varying intensity, mostly moderate to strong and some mild, and any staining was considered as positive staining. Representative formalin-fixed paraffin-embedded (FFPE) surgical resection specimen blocks of primary tumor from each case were obtained and randomly assigned to discovery (*n* = 44) or validation (*n* = 11) sets prior to the miRNA sequencing below. Sample assignment proportions, to discovery (80%) and validation (20%) sets, are in accordance with standard machine learning practices [29]. Our project was approved through the Research Ethics Board at Queen’s University (ethic code PATH-145-14, approved in 21 November 2019) and the Institutional Review Boards of Weill Cornell Medicine (ethic code 0406007186, approved in 18 February 2020) and The Rockefeller University (ethic code TTU-0707, approved in 22 May 2020). This is a study of de-identified tissues from the pathology department so there is no informed consent form.

### 2.2. Total RNA Isolation and Quality Control

Total RNA was isolated from two 1.5 mm tissue cores, bored from representative tumor-bearing blocks for each case, using the Qiagen RNeasy FFPE Kit (QIAGEN, Venlo, The Netherlands) according to the manufacturer’s guidelines. Total RNA concentrations and purity were determined using Qubit^®^ fluorometric quantitation (Thermo Fisher Scientific, Waltham, MA, USA).

### 2.3. Small RNA Sequencing

miRNA expression profiles were generated through quantitative barcoded small RNA sequencing as described [25,30]. Small RNA cDNA libraries were sequenced on an Illumina HiSeq 2500 platform (Illumina, San Diego, CA, USA) at the McGill University and Génome Québec Innovation Centre. FASTQ sequence files were subsequently demultiplexed and annotated through an established small RNA annotation pipeline, yielding individual miRNA, miRNA cistron, and calibrator expression data [24,31]. miRNA content was calculated as described [25]. Sequencing data are presented in Tables S1–S3.

### 2.4. Data Preprocessing

Data preprocessing and subsequent analyses were performed in MATLAB (Mathworks, Inc., Natick, MA, USA, version R2016b), as described in [25]. Briefly, data preprocessing comprised normalization, and outlier detection and removal through correlation analyses. Following preprocessing, all miRNA STAR sequences and non-human sequences were filtered. Additionally, only miRNAs expressed above the 95th percentile in more than 5% of samples from each tumor type were included in subsequent analyses. miRNA cistron expression data were similarly preprocessed.

### 2.5. High Expression and Discovery Analyses

To identify candidate miRNA tissue markers for lung NEN classification, high expression and discovery analyses were performed as described [25]. For high expression analyses, we identified the top 0.5% of expressed individual miRNAs and miRNA cistrons for all lung NENs, and then for each pathological type. For discovery analyses using discovery set profiles (*n* = 44), we used a novel feature selection algorithm with 5-fold validation [32] to rank individual miRNAs and miRNA cistrons that discriminate carcinoids from NECs. Briefly, the feature selection algorithm is an ensemble classifier that ranks the ability of each miRNA to discriminate between cancer types. Rankings are determined using average performance over fourteen established feature selection methods. Only the top-ranking 5% individual miRNAs and miRNA cistrons were used for classification below.

### 2.6. miRNA-Based Classifier for Discriminating Carcinoids from NECs

Using our machine learning approach [25], we constructed a miRNA-based classifier for discriminating carcinoids from NECs. After evaluating all available algorithms (*n* = 23) from the MATLAB Classification Learner App, we selected the linear discriminant algorithm for this classifier. Once established, we determined the accuracy of our classifier in the discovery and validation sets. To better understand the transferability of our classifier, we assessed the expression of individual miRNAs used for classification; miRNA cistrons were also examined to assess data consistency.

### 2.7. Candidate miRNA Markers for Discriminating Pathological Types

To identify candidate miRNA markers for discriminating TC from AC, and SCLC from LCNEC, we applied the same feature selection algorithm and ranking criteria as above. Due to limited sample size, we were unable to separate samples into discovery and validation sets; we instead identified candidate pathological type markers using all samples in a single cohort. After evaluating all available algorithms, we selected the Kernel Naïve Bayes algorithm to discriminate TC (*n* = 14) from AC (*n* = 15) and the Cosine k-nearest neighbours (KNN) algorithm to discriminate SCLC (*n* = 11) from LCNEC (*n* = 15).

### 2.8. Statistical Analyses

Statistical analyses of clinical data were performed using SPSS Statistics (IBM, Armonk, NY, USA, Version 25). Non-parametric Mann–Whitney U (MWW) or Kruskal–Wallis (K-W) tests were used to assess differences between two continuous variables [33]. Spearman correlation was used to measure correlation between variables [34]. Associations between categorical variables were analyzed using two-tailed Fisher’s exact test (FET) for 2 × 2 associations or the χ^2^ test for larger groups [35]; a two-tailed *p*-value of < 0.05 was considered statistically significant. These statistical tests were also used to correlate selected miRNA features (see Section 2.5) with Ki-67 staining (Spearman) and mitotic counts (Spearman), and to compare selected miRNA features between tumors with, and without, necrosis (MWW test), and with, and without, nodal metastases (MWW test). Only two patients were treated prior to tumor biopsy (one neoadjuvant chemotherapy, one DNA vaccine trial for a prior cancer), and only one patient had known distant metastasis at the time of diagnosis (i.e., stage 4); we were therefore unable to perform statistical analyses to evaluate miRNA changes associated with treatment.

## 3. Results

### 3.1. Clinicopathologic Characteristics of Discovery and Validation Sample Sets

The clinical characteristics and proportions of tumors were similar whereas pathologic characteristics varied by pathological type in discovery and validation sets. Age, gender, and other relevant clinicopathologic data are summarized in Table 1. No significant differences in age (MWW, U = 305.0, *p* = 0.958, *r* = −0.071) or gender (FET, χ^2^ = 0.024, df = 1, *p* = 0.877) were detected between sets. Similar proportions of TC, AC, SCLC, and LCNEC were present in each set (χ^2^ = 0.041, df = 3, *p* = 0.998). Ki-67 (K-W, H = 35.065, df = 3, *p* < 0.001), and mitotic counts (H = 39.291, df = 3, *p* < 0.001) were significantly different between pathological types in the discovery set; similar results for Ki-67 (H = 8.587, df = 3, *p* = 0.035) and mitotic counts (H = 9.495, df = 3, *p* = 0.023) were found in the validation set. We were unable to compare necrosis between pathological types due to low sample numbers.

### 3.2. Barcoded Small RNA Sequencing

Comprehensive miRNA expression profiles were generated for all samples through quantitative barcoded small RNA sequencing. Annotated sequence read counts are presented in Table S4; a median of 1,476,891 (range: 72,984–14,239,342) miRNA sequence reads, representing a median of 50.5% (range: 6.6–88.2%) total sequence reads, was obtained. Individual miRNA, miRNA cistron, and calibrator sequence read counts are presented in Tables S1–S3. Median miRNA content was 10.8 (range: 0.4–123.0) and 27.7 (range: 1.8–81.0) fmol per microgram total RNA per sample in discovery and validation sets, respectively; no significant differences in miRNA content were seen between pathological types in either set (K-W test, discovery: χ^2^ = 1.353, df = 3, *p* = 0.717; validation: χ^2^ = 6.242, df = 3, *p* = 0.100).

### 3.3. High Expression Analyses

**Figure 1:**
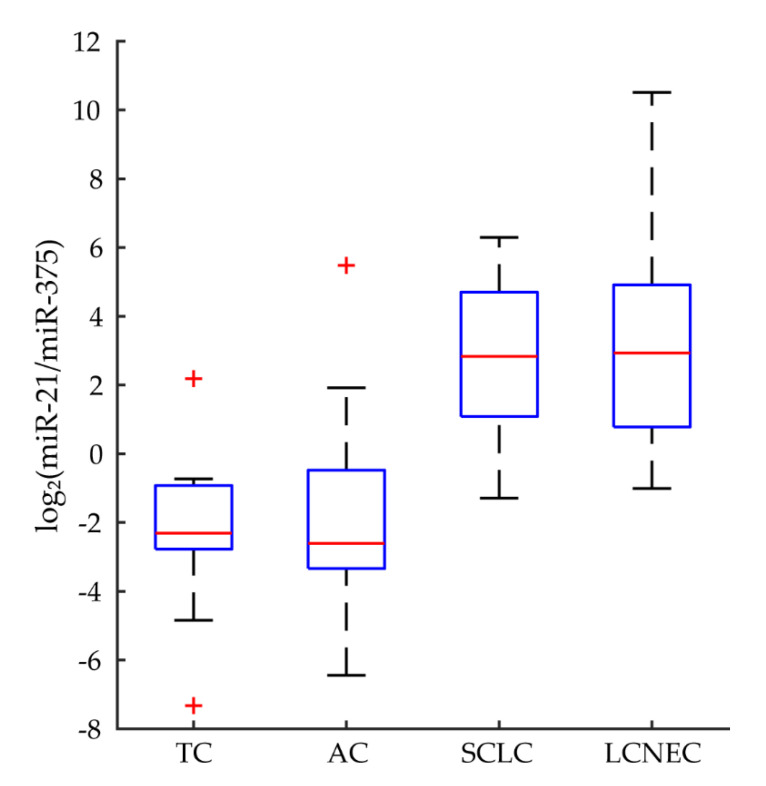
Ratio of miR-21 and miR-375 expression in lung NENs. Log_2_ transformed ratios of miR-21 and miR-375 expression were assessed in all samples. Ratios were significantly lower in carcinoids than NECs (MWW, U = 66.0, *p* < 0.001, *r* = −0.825). Red crosses denote statistical outliers. Abbreviations: typical carcinoid (TC), atypical carcinoid (AC), small cell lung carcinoma (SCLC), large cell neuroendocrine carcinoma (LCNEC).

Candidate miRNA markers for lung NENs were identified from the top 0.5% of expressed individual miRNAs and miRNA cistrons in all samples (*n* = 55, Table 2). miR-375, -21, -143, -141, let-7a, let-7f, -30d, and -148a were the highest expressed individual miRNAs, with median expression ranging from 2.0–8.2%. Clusters-miR-98(13), -miR-375(1), -miR-21(1), and -miR-143(2) were the highest expressed cistrons, with median expression ranging from 4.4–10.8%. We also identified the top 0.5% of expressed individual miRNAs and miRNA cistrons for each pathological type (Table S5). On further inspection, we observed that miR-21 expression was ranked lower in carcinoids than in NECs. Conversely, miR-375 expression was higher in carcinoids than in NECs. Log_2_ transformed ratios of miR-21 and miR-375 expression were significantly lower in carcinoids than NECs (MWW, U = 66.0, *p* < 0.001, *r* = −0.825) (Figure 1).

### 3.4. Discovery Analyses

Candidate miRNA markers that discriminate carcinoids from NECs were identified from the top-ranking 5% individual miRNAs (Table S6) and miRNA cistrons (Table S7), in our discovery set only. These rankings were used to build the miRNA-based classifier below.

### 3.5. miRNA-Based Classifier for Discriminating Carcinoids from NECs

**Figure 2:**
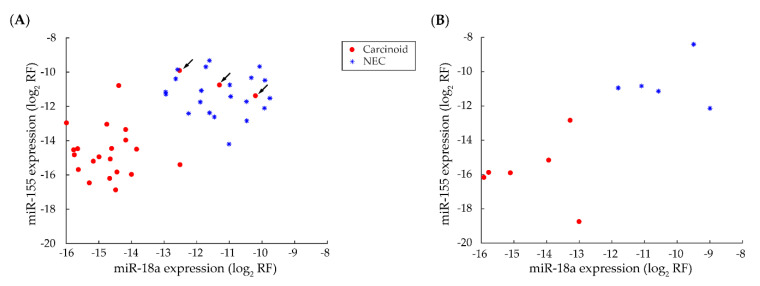
Scatter plot assessment of selected individual miRNAs for discriminating carcinoids from neuroendocrine carcinomas (NECs). Carcinoids and NECs were discriminated using miR-18a and -155, with three misclassifications in the discovery set (**A**) and no misclassification in the validation set (**B**). Abbreviation: log_2_ normalized relative frequency (log_2_ RF).

Using the linear discriminant algorithm, the highest performing algorithm for this comparison, we constructed a miRNA-based classifier for discriminating lung NENs using miR-18a and -155. Using these features, the classifier discriminated carcinoids from NECs with 93% and 100% accuracy in the discovery and validation sets, respectively (Figure 2 and Table 3). The median percentage of individual miRNA or miRNA cistron expression for selected miRNA markers ranged from 0.00–0.17% and 0.00–12.36%, respectively (Table S8). Based on our observation above, miR-21 and -375 were also evaluated. However, these features discriminated carcinoid from NEC with 86% accuracy in the discovery set, lower than miR-18a and -155.

### 3.6. Candidate miRNA Markers for Discriminating Pathological Types

Based on ranked feature selection, we identified miR-17, -103, and -127 as candidate markers to discriminate TC from AC, and miR-301a, -106b, and -25, as candidates to discriminate SCLC from LCNEC (Figure S1). Using these features, the selected algorithms (Kernel Naïve Bayes and Cosine k-nearest neighbors (KNN)) discriminated TC from AC in 29/29 (100%) cases, and SCLC from LCNEC, in 25/26 (96%) cases.

### 3.7. Correlation of Candidate miRNA Markers and Pathologic Parameters

Correlation analyses in the discovery set revealed the candidate biomarkers miR-18a, -155, -17, -127, -106b, and -25 are correlated with Ki-67 staining (Spearman’s rho = 0.785, 0.614, 0.788, −0.711, 0.685, 0.719, respectively; *p* < 0.05) and mitotic rate (Spearman’s rho = 0.670, 0.510, 0.694, −0.604, 0.641, 0.580; *p* < 0.05). Similar results were found in the validation set (Table S9). Pairwise comparisons of samples with necrosis, without necrosis, and with focal necrosis showed that miR-18a, -17, and -127 were differently expressed in all pairwise comparisons (MWW, *p* < 0.05), and miR-155, -106b, and -25 were differently expressed in two of three pairwise comparisons (MWW, *p* < 0.05). Comparisons between samples with, and without, necrosis were similar in the validation set; comparisons involving samples with focal necrosis in the validation set were not found to be significant. However, this may be because only one sample in the validation set had focal necrosis (Table S9). miR-103 and -301a were not significantly correlated with Ki-67 staining nor with mitotic rate, nor were they differently expressed in any pairwise comparisons of tumor necrosis. MWW tests showed that all candidate biomarkers were not differently expressed between tumors with, and without, nodal metastases (Table S9).

## 4. Discussion

Lung NEN classification conveys prognostic information and guides clinical management. Currently, lung NENs are classified based on morphological and cytological features, the presence or absence of necrosis, and immunoreactivity for markers of neuroendocrine differentiation [2,3]. However, accurate histologic evaluation can be impacted by sampling issues, uneven distribution of mitoses in tissue sections, misleading artifacts and/or confounding pathologic features, and challenges in identifying punctate necrosis or interpreting transitional cell characteristics [1]. To address the need for further research on lung NEN classification [3], we used our recently established sequence data mining approach to identify miRNA tissue markers that complement histologic evaluation [25].

The strength of our study stems from including all four pathological types of lung NEN in the same study, comprehensive miRNA detection from archived clinical samples [22,36], accurate sequence annotation [24], advanced computational approaches for ranked feature selection and classification [25,32], assessment of data reliability through knowledge of miRNA cistron composition [31], and accelerating transferability to other miRNA detection platforms by providing miRNA abundance data. In addition, molecular classification circumvents issues arising from histologic artifacts and/or confounding pathologic features.

High expression analyses indicated that miR-375, -21, -143, -141, let-7a, let-7f, -30d, and -148a are the most abundant individual miRNAs in lung NENs, accounting for approximately 30% of all miRNAs, in all samples. When analyzed by pathological type, we noticed that miR-21/-375 expression ratios are useful for discriminating low- and intermediate-grade from high-grade NENs. miR-21 is often upregulated in cancer and thought to be an oncogene [37,38], whereas miR-375 behaves like a tumor suppressor [39]; the regulatory roles of the other abundant individual miRNAs and miRNA cistrons in neuroendocrine tumorigenesis remain to be defined. The ratio of miR-21 and -375 may directly or indirectly reflect the balance of oncogenic and tumor suppressive activities in lung NENs. Despite the classificatory potential of this expression ratio, we found more accurate markers through feature selection below.

Discovery analyses enabled the identification of discriminating miRNA markers for lung NEN classification. Using our recently established method [25], we constructed and validated a classifier that accurately discriminates carcinoids from NECs, based on miR-18a and -155 expression. We also generated preliminary evidence for discriminating TC from AC, and SCLC from LCNEC, using miR-17, -103, and -127, and miR-301a, -106b, and -25, respectively. Despite accuracy rates of >90%, these findings require validation in prospective cohort studies, or external sample sets, due to limited sample size.

miRNAs selected for lung NEN classification also provide interesting pathomechanistic insights. miR-18a and -155 are more highly expressed and discriminate NECs from carcinoids. miR-18a is correlated with lung NEN aggression [17]; miR-155 has been previously identified as a discriminator between NECs and carcinoids [16], and likely reflects the number of hematopoietic cells admixed with the tumor sample [37]. miR-17 and -103 are less expressed and miR-127 more highly expressed in TC than AC, suggesting oncogenic and tumor suppressive roles, respectively. miR-301a, -106b, and -25 are more highly expressed in SCLC than LCNEC; given that both are high-grade tumors, these miRNAs more likely mediate tumor morphology than aggression. Correlation analyses suggest miR-18a, -155 -17, -127, -106b, and -25 are related to Ki-67 expression and mitotic rate; differential expression analyses suggest they may also be related to necrosis, however we were unable to validate these findings due to low sample size. As these pathologic parameters are all significantly different in TC, AC, SCLC, and LCNEC, further investigation is required to elucidate the functional roles of these miRNAs.

Our current study has similar limitations to our published study on miRNA-based gastroenteropancreatic NEN classification [25]. Assembling large collections of rare tumor samples is challenging, functional imaging and pathologic data are often not linked, assessing the prognostic value of candidate miRNA markers may not be possible due to uneven clinical follow-up, and comparing results between studies can be challenging due to inherent differences between miRNA detection methodologies [13]. Nonetheless, we continue to build knowledge of miRNA expression in NENs that can be leveraged by clinical and basic investigators.

We have developed and validated a miRNA-based classifier for discriminating carcinoids from NECs, provided candidate miRNA markers for differentiating pathological types, shown potential for identifying aggressive AC cases through miRNA expression ratios, and provided comprehensive reference miRNA profiles to stimulate further investigation. Our research directions include additional miRNA profiling of well annotated lung NEN sample collections, and functional characterization of selected miRNAs in neuroendocrine tumorigenesis.

## 5. Conclusions

Combined molecular and machine learning methods have much promise for accurate tumor classification. Using a representative approach, we have developed, and internally validated, a simple miRNA-based classifier, comprising miR-18a and -155, to discriminate low-grade carcinoids from high-grade NECs, with a high degree (>90%) of accuracy. We have also identified miR-17, -103, and -127 as candidate markers to discriminate TC from AC, and miR-301a, -106b, and -25, as candidates to discriminate SCLC from LCNEC. To fully explore the clinical utility of these markers, future studies should incorporate larger numbers of well-annotated clinical samples.

## Figures and Tables

**Table 1:** Relevant clinical and pathologic data for the four pathological types of lung neuroendocrine neoplasm (NEN) included in discovery and validation sets.

Features	Discovery Set				Validation Set			
	Carcinoids		NECs		Carcinoids		NECs	
	TC (*n* = 11)	AC (*n* = 12)	SCLC (*n* = 9)	LCNEC (*n* = 12)	TC (*n* = 3)	AC (*n* = 3)	SCLC (*n* = 2)	LCNEC (*n* = 3)
Male:female	1:10	2:10	4:5	4:8	0:3	1:2	1:1	1:2
Age avg (min, max)	64 (41, 85)	61 (43, 79)	67 (50, 86)	69 (45, 85)	64 (50, 74)	60 (54, 64)	69 (65, 73)	71 (67, 73)
Tumor size avg in mm (min, max)	18 (5, 30)	25 (12, 66)	27 (11, 70)	28 (10, 70)	12 (3, 22)	28 (4, 43)	26 (18, 35)	26 (15, 32)
Ki-67 avg (min, max)	1 (<1, 3)	5 (<1, 38)	61 (33, 73)	27.5 (7, 51)	<1 (<1, 3)	<1 (<1, 5)	56 (53, 59)	69 (62, 75)
Mitosis avg (min, max)	0.3 (0, 1.3)	3.9 (2, 18)	88 (49, 183)	27 (11, 85.3)	0.3 (0, 1.3)	2 (2, 3)	80 (63, 97)	42.7 (39, 60)
Necrosis (yes, no, focal)	0, 11, 0	0, 4, 8	9, 0, 0	12, 0, 0	0, 3, 0	0, 3, 0	2, 0, 0	2, 0, 1
pT category								
1	10 (91%)	8 (67%)	6 (67%)	4 (33%)	3 (100%)	1 (33%)	1 (50%)	1 (33%)
2	0 (0%)	4 (33%)	2 (22%)	8 (67%)	0 (0%)	2 (67%)	1 (50%)	2 (67%)
3	0 (0%)	0 (0%)	1 (11%)	0 (0%)	0 (0%)	0 (0%)	0 (0%)	0 (0%)
4	1 (9%)	0 (0%)	0 (0%)	0 (0%)	0 (0%)	0 (0%)	0 (0%)	0 (0%)
pN category								
Unknown	1 (9%)	0 (0%)	1 (11%)	1 (8%)	1 (33%)	0 (0%)	0 (0%)	0 (0%)
0	8 (73%)	9 (75%)	5 (56%)	5 (42%)	2 (67%)	3 (100%)	2 (100%)	2 (67%)
1	2 (18%)	1 (8%)	1 (11%)	4 (33%)	0 (0%)	0 (0%)	0 (0%)	1 (33%)
2	0 (0%)	2 (17%)	2 (22%)	2 (17%)	0 (0%)	0 (0%)	0 (0%)	0 (0%)
Inter-set comparison	TC		AC		SCLC		LCNEC	
Gender	χ2 = 0.029, df = 1, *p* = 0.588		χ2 = 0.417, df = 1, *p* = 0.519		χ2 = 0.020, df = 1, *p* = 0.887		χ2 = 0.000, df = 1, *p* = 1.000	
Age	U = 15.0, *p* = 0.863, *r* = −0.063		U = 14.5, *p* = 0.664, *r* = −0.131		U = 8.0, *p* = 0.909, *r* = −0.071		U= 17.5, *p* = 0.966, *r* = −0.019	

Gender and age differences are evaluated with Fisher’s exact and Mann–Whitney U tests, respectively; mitosis is presented as mitotic figures per 2 mm^2^. Abbreviations: neuroendocrine carcinomas (NECs), typical carcinoid (TC), atypical carcinoid (AC), small cell lung carcinoma (SCLC), large cell neuroendocrine carcinoma (LCNEC), average (avg), pathologic tumor category (pT), pathologic nodal category (pN), degrees of freedom (df).

**Table 2:** Median expression of the top 0.5% overall highest expressed microRNAs (miRNAs) and miRNA cistrons.

miRNA	Median % of miRNA in all Samples
miR-375	8.2
miR-21	7.9
miR-143	4.1
miR-141	2.9
let-7a	2.9
let-7f	2.5
miR-30d	2.4
miR-148a	2.0
**miRNA Cistron**	**Median % of miRNA Cistron in all Samples**
cluster-mir-98(13)	10.8
cluster-mir-375(1)	8.2
cluster-mir-21(1)	7.9
cluster-mir-143(2)	4.4

Cluster-mir-98(13) comprises miR-125b-1, let-7a-2, miR-100, miR-99a, let-7c, miR-125b-2, let-7a-3, let-7b, let-7a-1, let-7f-1, let-7d, miR-98, and let-7f-2; clusters-mir-375(1) and -mir-21(1) are monocistronic; and cluster-mir-143(2) contains miR-143 and miR-145.

**Table 3:** Overall accuracy of the miRNA-based classifier for discriminating lung NENs.

Hierarchical Classifier Designation	Pathologic Diagnosis			
	Discovery Set		Validation Set	
	Carcinoids	NECs	Carcinoids	NECs
Carcinoids	20	0	6	0
NECs	3	21	0	5
Overall accuracy	41/44 (93%)		11/11 (100%)	

## Supplementary Materials

The following are available online at https://www.mdpi.com/2072-6694/12/9/2653/s1, Figure S1: Scatter plot assessment of candidate markers for discriminating lung NEN pathological types, Table S1: Individual miRNA sequence read counts for all study samples, Table S2: miRNA cistron sequence read counts for all study samples, Table S3: Calibrator sequence read counts for all study samples, Table S4: Small RNA sequencing annotation statistics for all study samples, Table S5: High expression analyses of miRNA profiles for each pathological type, Table S6: Top-ranked discriminatory miRNAs identified through feature selection, Table S7: Top-ranked discriminatory miRNA cistrons identified through feature selection, Table S8: Median percentage of individual miRNA expression for selected classificatory markers, Table S9: Candidate miRNA biomarker expression in relation to pathologic features.
