# Supplementary material for: Classifying Lung Neuroendocrine Neoplasms through MicroRNA Sequence Data Mining
Source: Cancers (Basel). 2020 Sep 17;12(9):2653. doi: 10.3390/cancers12092653 (PMC7564332; doi:10.3390/cancers12092653)
Supplement: Supplementary file 1 [file cancers-12-02653-s001.zip › cancers-946085 Supplementary Material.pdf]

Supplementary Material: Classifying Lung Neuroendocrine Neoplasms through MicroRNA Sequence Data Mining

Justin J.M. Wong, Paula S. Ginter, Kathrin Tyryshkin, Xiaojing Yang, Jina Nanayakkara, Zier Zhou, Thomas Tuschl, Yao-Tseng Chen and Neil Renwick

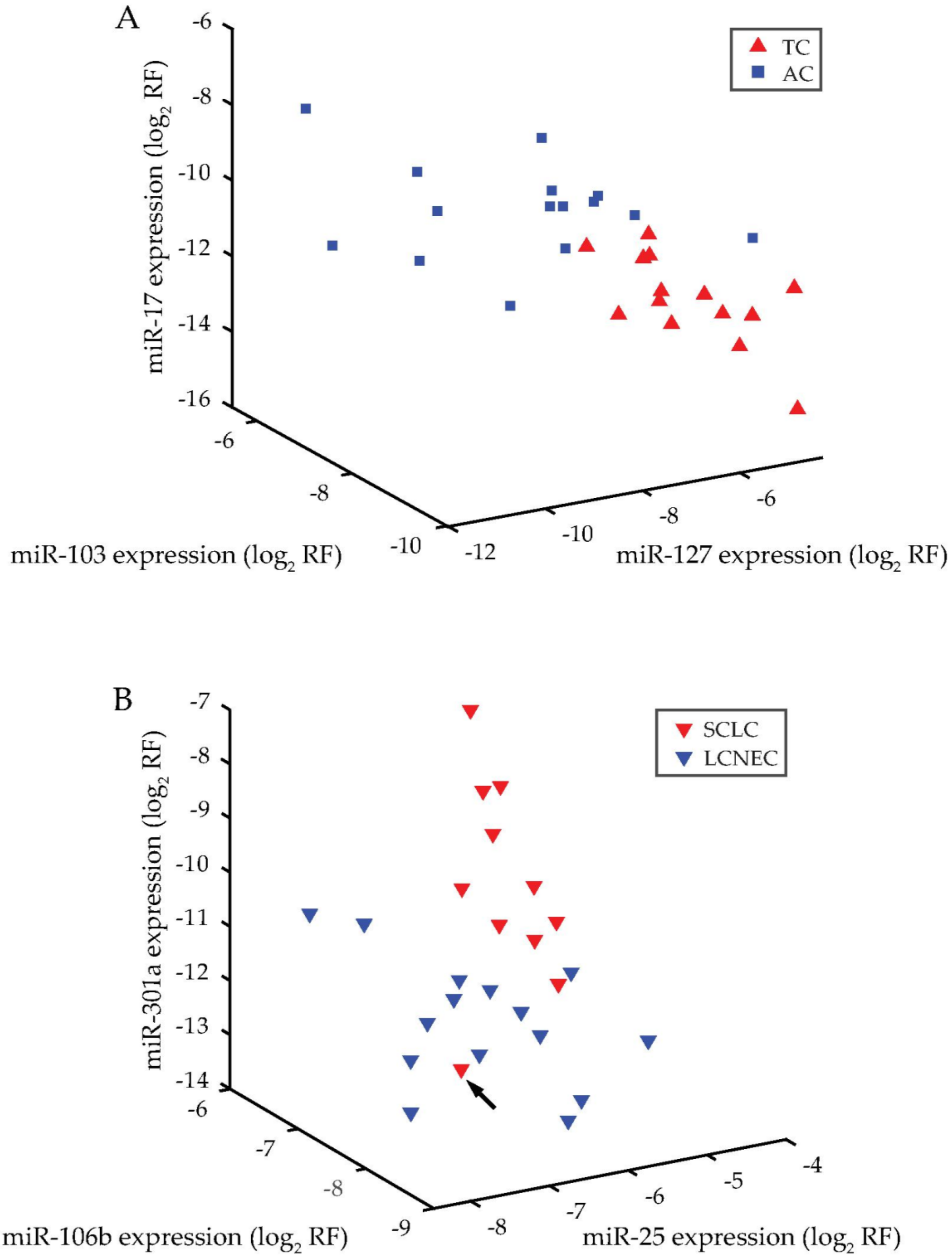

Figure S1. Scatter plot assessment of candidate markers for discriminating lung NEN pathological types. TC and AC were discriminated using miR-17, 103 and -127 with no misclassification (**A**). SCLC

and LCNEC were discriminated using miR-301a, -106b, and -25 with one misclassification (**B**). Abbreviation: $\log_2$ normalized relative frequency ($\log_2$ RF).

Table S1 (separate Excel file): Individual miRNA sequence read counts for all study samples. Annotated human and viral miRNA sequence read counts are presented for all samples in discovery and validation sets.

Table S2 (separate Excel file): miRNA cistron sequence read counts for all study samples. Annotated human and viral miRNA cistron sequence read counts are presented for all samples in discovery and validation sets; the number of members in each miRNA cistron is indicated in brackets following the cistron name e.g., cluster-hsa-mir-1-1(4).

Table S3 (separate Excel file): Calibrator sequence read counts for all study samples. Annotated calibrator sequence reads are presented for all samples in discovery and validation sets; the calibrator cocktail consisted of ten different oligoribonucleotide sequences.

Table S4 (separate Excel file): Small RNA sequencing annotation statistics for all study samples. Small RNA sequence read counts and proportions for all annotation categories including calibrators, ribosomal rRNA (rRNA), snoRNA (small nucleolar RNA), small nuclear RNA (snRNA), transfer RNA (tRNA), other (miscellaneous low abundance RNAs), and microRNA (miRNA) are presented for all small RNA profiles in discovery and validation sets. miRNA content (fmol per microgram total RNA) per sample is calculated as described [20].

Table S5 (separate Excel file): High expression analyses of miRNA profiles for each pathological type. The median expression of the top 1% of individual miRNAs and miRNA cistrons for carcinoid and NEC gruoups, and for each pathological type, namely TC, AC, SCLC is presented in descending order.

Table S6 (separate Excel file): Top-ranked discriminatory miRNAs identified through feature selection. The top 3% candidate individual miRNA markers for discriminating (**A**) carcinoids from NECs, (**B**) TC from AC, and (**C**) SCLC from LCNEC are presented in ranked descending order. Comparison A rankings were generated using discovery set samples (n = 44) whereas comparison B and C rankings were generated using all study samples (n = 55). Sequence read counts for each individual miRNA are presented as normalized median expression per million. Overall rank indicates average ranking over 5-fold validation.

Table S7 (separate Excel file): Top-ranked discriminatory miRNA cistrons identified through feature selection. The top 3% candidate miRNA cistron markers for discriminating (**A**) carcinoids from NECs, (**B**) TC from AC, and (**C**) SCLC from LCNEC are presented in ranked descending order. Comparison A rankings were generated using discovery set samples (n = 44) whereas comparison B and C rankings were generated using all study samples (n = 55). Sequence read counts for each individual miRNA are presented as normalized median expression per million. Overall rank indicates average ranking over 5-fold validation.

Table S8 (separate Excel file): Median percentage of individual miRNA expression for selected classificatory markers. Normalized individual miRNA read counts in discovery and validation sets are presented for miRNAs selected for NEN classification. Knowledge of miRNA abundance facilitates comparisons with other miRNA detection methods.

Table S9 (separate Excel file): Candidate miRNA biomarker expression in relation to pathologic features. Candidate biomarker expression was evaluated for (**A**) correlation to Ki-67 and mitotic rate, (**B**) differential expression between samples with necrosis, without necrosis, and with focal necrosis, and (**C**) differential expression between samples with and without nodal metastases. As there were no N2 samples in the validation set, comparisons involving this stage could not be performed. Medians are presented as $\log_2$ normalized relative frequency. Abbreviation: Not Applicable (NA).
